# Microsatellite instability in colorectal cancer

**DOI:** 10.2478/v10019-011-0005-8

**Published:** 2011-03-15

**Authors:** Matej Horvat, Borut Stabuc

**Affiliations:** 1 University Medical Centre Maribor, Maribor, Slovenia; 2 Department of Gastroenterology, University Medical Centre Ljubljana, Slovenia

**Keywords:** colorectal cancer, microsatellite instability, chemotherapy

## Abstract

**Background:**

Colorectal cancer (CRC) is the third most common cancer in the world. In 75% CRC develops sporadically, in 25% hereditary or as a consequence of inflammatory bowel disease. CRC carcinogenesis develops over many years. The cause of CRC in 85% is chromosomal instability (CIN) and in 15% microsatellite instability (MSI-H), where hereditary nonpolyposis colorectal cancer (HNPCC) represents 10–20%. Microsatellite sequences (MS) are repeated sequences of short stretches of DNA all over the genome. Microsatellite stability (MSS) means MS are the same in each cell of an individual, whereas microsatellite instability (MSI-H) means MS differ in normal and cancer cells of an individual. The cause of MSI-H is a damaged mismatch repair mechanism (MMR), with the most important MMR proteins being MSH2, MLH1 and MSH6.

**Conclusions:**

MSI-H seems to be an important prognostic factor in CRC and an important predictive factor of CRC chemotherapeutic treatment efficacy. Clinical trials conducted until now have shown contradictory findings in different chemotherapeutic settings, adjuvant and palliative; therefore MSI-H is going to be the object of the future research. The future of cancer treatment is in the individualized therapy based on molecular characteristics of the tumour, such as MSI-H in CRC.

## Introduction

Colorectal cancer (CRC) is the third most common cancer and the fourth most common cause of cancer related deaths in the world.^1^ CRC incidence in last decades is steadily growing.^2^ CRC incidence in Slovenia in 2007 was 1392 new cases. It was the third most common cancer in males and second most common cancer in females.^3^ CRC is a significant public health problem.^4^ CRC develops in 75% sporadically because of mutations acquired during a person’s lifetime and in 25% as a combination of hereditary syndromes, a higher risk because of CRC familial burden without criteria for a hereditary syndrome or as consequence of inflammatory bowel disease (IBS).^1^,^5^

## Colorectal cancer carcinogenesis

20 years ago Fearon and Vogelstein developed a theory about CRC carcinogenesis on the genetical level they called multistep carcinogenesis.^6^ With this theory they explained the progress of normal colon and rectum mucosa through adenomas to malignant growth. A normal balance of mucosa cells in colon and rectum is maintained by their origin in the colonic crypt, their migration to the surface epithelia and finally apoptosis in the surface epithelia. This process reverts in adenomatous polyps and in malignant growth. There is less apoptosis in the surface epithelia and more in the colonic crypt, both is proportional to the level of malignancy. Mucosa cells become more susceptible to DNA damage, DNA methylation and reverse levels of apoptosis.^7^ CRC carcinogenesis is promoted by mutations in genes involved in cellular differentiation, mitosis, growth and cellular death.^8^ CRC cancerogensis is a process that lasts 5–10 years. With presence of malignant growth the quantity of genetic mutations potentiates.^9^,^10^ The growth of CRC from a local to a disseminated form lasts further 3–5 years.^11^

CRC develops because of the genomic instability as a consequence of mutations in gatekeeper and caretaker genes.^12^ There are two forms of genomic instability: chromosomal instability (CIN) and microsatellite instability (MSI).^8^,^13^ CIN represents 85% of genomic instability. CIN develops because of chromosomal translocations, rearrangements of parts of chromosomes and gene multiplication.^14^,^15^ CIN develops in genes participating in chromosomal condensation, centrosome and microtubule formation and cell cycle checkpoints.^16^ CRC developing through CIN pathway is aneuploid. The most common affected genes in CIN are protooncogene KRAS, tumour suppressor genes APC and p53 and BUB family genes that regulate cell cycle.^17^,^18^

## Microsatellite instability

CRC developing because of MSI has smaller genomic abnormalities, CRC is diploid without major chromosomal abnormalities.^19^ Present are point mutations, substitutions, insertions or deletions of one or a smaller number of nucleotides.^20^ Microsatellite sequences (MS) are repeating stretches of DNA located throughout the entire genome: intronic parts of genes, gene promotors, untranslated terminal regions and exonic parts of genes.^21^ MS are one to six base pairs long and are repeated many thousand times.^13^ MS are identical in each cell of an individual, normal and malignant, condition referred to as microsatellite stability (MSS). MSI is a condition where MS differ in normal and malignant cells of an individual.^22^ MSI is defined according to the presence of five Bethesda markers, three of them are dinucleotide markers (D2S123, D5S346 and D17S250) and two of them are mononucleotide markers (BAT25 in BAT26). There is an arbitrary agreement that MSI is present if normal and malignant cells of an individual differ in at least one of the Bethesda markers. MSI high (MSI-H) is present if they differ in at least two of the markers and MSI low (MSI-L) is present if they differ in one of the markers.^23^ MS are susceptible to insertions or deletions at the point of replication. Replication is a process requiring the highest level of fidelity, because a replication error might induce mutations in every daughter cell.^24^ The fidelity of replication is ensured by complementarities of nucleotide base pairs and the enzyme DNA polymerase with its proofreading activity. They reduce the possibility of mismatched base pairs to one in one million. With the size of human genome being 3×10^9^ base pairs the rate of mutation would be more than thousand errors with each cell replication.^25^ Because of this, human cells need another proofreading mechanism enabling the highest fidelity of replication. This mechanism is called mismatch repair mechanism (MMR). An intact MMR lowers the rate of mutation for another one hundred to six hundred times.^26^

In cells with MMR genes mutation replication errors occur, MS develop mutations and in some cells MSI-H occurs. MSI-L CRC does not appear to differ clinically or pathologically from MSS CRC.^27^ The lack of an intact MMR mechanism is a cause of the tumour suppressor gene inactivation and of the occurrence of either sporadic or hereditary CRC. The hereditary form of CRC developing in this manner is hereditary nonpolyposis colorectal cancer (HNPCC) - Lynch syndrome and it represents 1–3% of all CRC incidence.^28^ The other form of MSI-H related CRC with the lack of intact MMR mechanism develops sporadically without hereditary mutations. The cause of this are epigenetic changes in the genome, CpG promoter hipermethylation of MMR genes, lowering the rate of their expression.^29^,^30^ The consequence are base pair insertions or deletions and frame shift mutations.^21^ MSI-H also effects TGFßRII gene mutation, a gene participating in cell signalization, and BAX gene mutation, a gene participating in the apoptosis regulation.^31^ Sporadic forms of CRC develop in this manner in approximately 15%. MSI-H is a cause of some other cancers; it affects the development of 5% of endometrial, ovarian and stomach cancer.^32^

**FIGURE 1.**
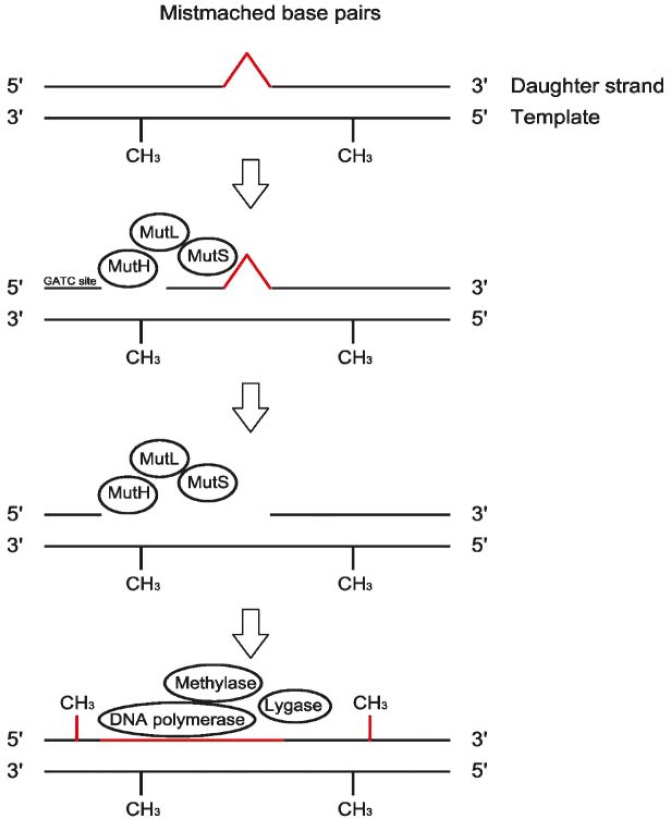
Mismatch repair. MutS protein binds to mismatched base pairs. MutL and MutH bind to the complex. MutH recognizes the daughter DNA strand which is not methylated, splits it at nearest GATC sites and excises the DNA strand. DNA polymerase, ligase and methylase complete the daughter strand.

The functions of MMR proteins are recognition of mis-incorporated base pairs, the recognition of mother and daughter DNA strand and repairing mis-incorporated base pairs with the base excision. In bacteria MMR mechanism is comprised out of three MMR proteins: MutS, MutL and MutH (Figure 1).^33^

MutS protein forms a dimmer and recognizes mismatched base pairs on the daughter strand or nucleotides not being paired. MutL protein binds to the MutS - the daughter DNA strand complex and enables binding of MutH protein. MutH recognizes the daughter DNA strand that is not methylated. The daughter strand is split at the nearest GATC sites in 5′ and 3′ direction. MutH also has an endonuclease activity; it excises the daughter DNA strand between the both restriction sites. DNA polymerase and ligase complete the missing daughter strand and enzyme methylase finishes the process of replication by methylating it.^34^ MMR mechanism is a process that has been highly conserved during the evolution from bacteria to human, the latter being far more complex. Each bacterial Mut protein has many human homologs. Bacterial MutS protein has three human homologs: hMSH2, hMSH3 and hMSH6. Bacterial MutL protein has also three human homologs: hMLH1, hPMS1 and hPMS2. Human homologs of bacterial MutH protein have not been discovered yet, their function is performed by MutL homologs.^35^,^36^

In hereditary form of CRC HNPCC most commonly mutated genes are hMSH2, hMSH6 in hMLH1. In the sporadic form of CRC the most commonly present mutation is an epigenetic alteration, hMLH1 promotor hypermethylation, and its subsequent lower expression.^29^ MSI-H is an early event in CRC carcinogenesis, it is present in 57% of HNPCC adenomas and in 3% of sporadic adenomas.^37^

## Pathohistological characteristics of MSI-H colorectal cancer

CRC is defined as MSI-CRC because of mutations present in the MS of CRC cancer cells. MSI-H and MSI-L are further characterized by the number of positive Bethesda markers as noted earlier in this article. CRC is defined as being MSS if there are no mutations in the MS of CRC cancer cells. Apart from its genetic origin MSI-H CRC differs from MSS CRC in many other features.^38^ MSI-H CRC is in a big proportion of 86–100% located proximally to the splenic flexure, MSS CRC is located there in 25% of cases.^39^,^40^ Upon the pathohistological examination MSI-H tumours are poorly differentiated, mucinous and have an intensive lymphocytic infiltration in the region surrounding the tumour in comparison to MSS tumours.^38^,^40^ MSI-H tumours have a larger primary local mass.^41^ MSI-H tumours have a better prognosis.^42^ MSI-H tumours rarely metastize.^43^ MSI-H tumours develop from hyperplastic adenomas with already present mutations and lower expression of hMLH1 gene.^38^ MSI-H tumours have a highly homogenous cell population.^43^ Better MSI-H CRC prognosis is attributed to a high proportion of mutations that act self destructively on tumour cells and cause the further mutation in genes the cell needs for its survival. Mutated proteins may also incorporate in the cell membrane of MSI-H tumour cells causing an immune reaction by the organism and the destruction of the tumour cell; a fact that may also explain the intensive lymphocytic infiltration in MSI-H tumours.^41^ Cell line trials conducted on CRC tumour cells have shown that MSI-H tumour cells next to all its genetic and patomorphological differences, also have characteristics of a predictive factor for the chemotherapeutic treatment efficacy. Trials conducted on cell lines have shown resistance of cell lines with defective MMR system and MSI-H to some chemotherapeutic regimens.^38^,^44^–^48^

## MSI-H as a prognostic factor in colorectal cancer and predictive factor in colorectal cancer chemotherapy

The chemotherapeutic treatment carries with its adverse effects that are more or less expressed in an individual.^49^ Clinical trials currently conducted are trying to elucidate the efficacy of the treatment and also the causes of chemotherapeutic regimens toxicity.^50^ The chemotherapeutic treatment is effective in a certain proportion of patients; with disseminated CRC the treatment response to 5-FU regimens is 20–25%, in combination of 5-FU with novel chemotherapeutics irinotecan and oxaliplatin the response rate is 45–50%.^51^ This means that chemotherapy is not only ineffective, but also causes many adverse effects for a large group of patients with no benefit.^52^ Clinical trials wish to elucidate predictive factors for the chemotherapeutic treatment and predictive factors for adverse effects, that could be used in everyday clinical setting. Cancers in the same stage of the TNM staging system according to their clinicopathological characteristic, differ in their clinical course, because of heterogeneity of their molecular characteristics.^42^ Toxicity of chemotherapeutics is influenced by patient comorbidity and by individual molecular variability.^50^ One of the potential predictive factors for the chemotherapeutic treatment efficacy and for the adverse effects level in an individual is MSI-H.^50^,^52^ Many clinical trials about MSI-H as a prognostic factor for CRC and as a predictive factor with adjuvant and palliative CRC chemotherapy have been conducted in last ten years.

Three classes of chemotherapeutic agents are used in CRC treatment: antimetabolites, alkylating agents and topoisomerase inhibitors.^29^,^38^ Antimetabolite used is called 5-flourouracil (5-FU). 5-FU is converted in the cell in two active forms that affect RNA synthesis and enzyme thymidylate syntethase (TS).^53^ Enzyme TS induces synthesis of thymidine monophosphates, 5-FU inhibits its action. MSI-H tumours cells (with defective MMR system) do not recognize the mutations caused by 5-FU on the DNA strand and they do not induce apoptosis.^54^,^55^ The resistance of MSI-H cell lines to 5-FU is explained by the fact that 5-FU sensitivity depends on the effective MMR system to induce apoptosis. *In vitro* cell line trials concerning MSI-H cells have shown the resistance to the treatment with 5-FU.^56^,^57^ Clinical trials concerning MSI-H patients and 5-FU treatment have shown contradicting results.^30^,^58^ The first clinical trial of MSI-H patients with adjuvant chemotherapy (5-FU monotherapy) has shown a better survival for patients receiving adjuvant chemotherapy, but clinical trials following have not shown that benefit.^41^,^59^

Chemotherapeutic irinotecan causes with inhibition of enzyme topoisomerase I brakes of DNA strand and apoptosis of cancer cell. *In vitro* cell line trials concerning MSI-H cells have shown higher sensitivity to irinotecan.^46^,^60^ Clinical trials have confirmed those results.^38^,^48^,^61^

Chemotherapeutic oxaliplatin is an alkylating agent and is a platinum analog. Platinum analogs form covalent bonds with DNA strand stopping the cell cycle and causing apoptosis.^62^,^63^ Cell lines with defective MMR system have a lower sensitivity to platinum analogs, because there is no effective MMR system to recognize DNA strand defects and induce apoptosis.^44^–^47^

In the recent 5 years several metaanalyses concerning MSI-H as a prognostic and predictive factor of CRC chemotherapeutic treatment were performed (Table 1). In 2005 Popat *et al.* have conducted the first metaanalysis of clinical trials about MSI-H as a prognostic factor in CRC.^42^ Metaanalysis included 32 clinical trials with 7642 patients, 1277 of them were MSI-H, representing 16.7% of all patients. The conclusion of the metaanalysis was that patients with MSI-H have a better survival than MSS patients in the same stage of the disease. In 2009 Des Guetz *et al.* have conducted a metaanalysis of 7 clinical trials of MSI-H as a predictive factor in adjuvant chemotherapeutical setting in stage II and III of the disease after the surgical treatment.^64^ Metaanalysis included 7 clinical trials with 3690 patients, 454 of them were MSI-H, representing 14% of all patients. Patients received adjuvant 5-FU based chemotherapy. MSI-H patients receiving adjuvant chemotherapy did not have a better survival than MSI-H patients not receiving chemotherapy. MSS patients receiving adjuvant chemotherapy had a better survival than MSS patients not receiving adjuvant chemotherapy. These results show an appearance of chemoresistance of MSI-H patients to adjuvant 5-FU based chemotherapy. In 2009 Des Guetz *et al.* have also conducted a metaanalysis of 6 clinical trials of MSI-H as a predictive factor in palliative chemotherapeutical setting in stage IV of the disease.^65^ Metaanalysis included 6 clinical trials with 964 patients, 91 of them were MSI-H, representing 9.4% of all patients. The conclusion of the metaanalysis was that patients with MSI-H have a statistically significantly better survival than MSS patients in the same stage of the disease. The efficacy of the chemotherapeutical treatment did not differ in MSI-H and MSS patients in five trials, in one of the trials MSI-H patients had a better survival than MSS patients.^30^ In one of the trials the better efficacy of higher doses of chemotherapy was observed among MSI-H patients.^66^ Both metaanalyses by Des Guetz included clinical trials with chemotherapeutical regimens that differed from each other, which made it difficult to objectively compare the results. From the results we conclude that there is an appearance of chemoresistance of MSI-H patients to adjuvant 5-FU based chemotherapy, making MSI-H a negative predictive factor for 5-FU adjuvant chemotherapy. In metastatic setting there was no clear conclusion about MSI-H as a predictive factor. The incidence of MSI-H in stages II and III was higher than in metastatic setting. In 2010 Guastadisegni *et al.* have conducted a metaanalysis of 31 clinical trials with 12872 patients, 1972 of them were MSI-H, representing 15.4% of all patients in all stages of the disease.^67^ The conclusion of the metaanalysis regarding MSI-H as a prognostic factor was the same as in the previous metaanalysis that patients with MSI-H have a better survival than MSS patients in the same stage of the disease. Metaanalysis included clinical trials with chemotherapeutical regimens that differed from each other, what made it difficult to objectively compare results and to come to a clear conclusion about MSI-H as a predictive factor. The authors concluded that regarding the complexity of 5-FU in the treatment of CRC MSI-H was only one of the important predictive factors that functioned with others that still had to be elucidated.

CRC develops through MSI-H pathway in 15%. It, therefore, would not be cost efficient to determine the MSI-H status in each CRC patient. In 2010 Sinicrope *et al.* have conducted a trial where they developed a prognostic model of determining probability of MSI-H in CRC regarding the clinical and pathological characteristics of patients diagnosed with CRC.^68^ When the tumour is proximally localized, poorly differentiated and when the patient is female, there is a 51% probability of MSI-H CRC incidence in comparison to 15% MSI-H CRC in general CRC population. When they considered lymphocytic infiltration the probability got even higher. Using this prognostic model it would make determining MSI-H status more cost efficient.

## Conclusions

Cancer patients nowadays have more and more diagnostic and therapeutic possibilities. The future of their treatment is an individualized therapy determined by patient characteristics and by tumour molecular characteristics that influence survival, chemotherapeutic treatment efficacy and incidence of adverse effects. MSI status is one of those prognostic and predictive factors in CRC.

## Figures and Tables

**TABLE 1.** Summary of metaanalysis performed concerning MSI-H as a prognostic and predictive factor in CRC

**Metaanalysis (year)**	**Number of clinical trials/Number of patients**	**Patients with MSI-H/%**	**Conclusion**
Popat *et al.* (2005)	32/7642	1277/16,7 %	MSI-H a positive prognostic factor
DesGuetz *et al.* (2009) Adjuvant chemotherapy setting	7/3690	454/14,0 %	MSI-H a negative predictive factor in adjuvant setting
DesGuetz *et al.* (2009) Metastatic chemotherapy setting	6/964	91/9,4 %	MSI-H a positive prognostic factor. No clear conclusion concerning MSI-H as a predictive factor
Guastadisegni *et al.* (2010)	31/12872	1972/15,4 %	MSI-H a positive prognostic factor. No clear conclusion concerning MSI-H as predictive factor, due to trial heterogenity
